# Drivers of household antibiotic use in urban informal settlements in Northern Ghana: Implications for antimicrobial resistance control

**DOI:** 10.1002/hsr2.1388

**Published:** 2023-06-29

**Authors:** Ezekiel K. Vicar, Williams Walana, Augustina Mbabila, George K. Darko, Kwame Opare‐Asamoah, Saeed F. Majeed, Mauvina Obeng‐Bempong

**Affiliations:** ^1^ Department of Clinical Microbiology University for Development Studies Tamale Ghana; ^2^ Nursing and Midwifery Training College Zuarungu Ghana; ^3^ Ghana Health Service Tolon District Hospital Tolon Ghana; ^4^ Department of Biological Sciences University for Development Studies Tamale Ghana; ^5^ Department of Pediatrics and Child Health Tamale Teaching Hospital Tamale Ghana

**Keywords:** antibiotic resistance, informal settlements, Tamale, Ghana

## Abstract

**Background:**

Urban informal settlements have been described as the epicenters of frequent antibiotic misuse, which has local and global consequences on the goals of antimicrobial stewardship. The aim of this study was to assess the relationship between knowledge, attitude, and practices of antibiotic use among households in urban informal settlements in the Tamale metropolis of Ghana.

**Method:**

This study was a prospective cross‐sectional survey of the two major informal settlements in the Tamale metropolis, namely Dungu‐Asawaba and Moshie Zongo. In all, 660 households were randomly selected for this study. Households with an adult and at least a child under 5 years old were randomly chosen. An adult with knowledge of household healthcare practices was selected  to respond to a structured questionnaire.

**Results:**

In all, 291 (44.1%) of the 660 households reported taking at least one type of antibiotic within the last month before the study and 30.9% (204/660) had used antibiotics without a prescription. Information on which antibiotics to use was obtained mostly from friends/family members 50 (24.5%) and were commonly purchased from a medical store or a pharmacy 84 (41.2%), saved up from a previously used antibiotic 46 (22.5%), a friend/family members 38 (18.6%), and drug hawkers 30 (14.7%). Amoxicillin 95 (26.0%) was the most frequently used antibiotic and the commonest indication for antibiotics use was diarrhea 136 (37.9%). Female respondents (odds ratio [OR] = 3.07; 95% confidence interval [CI] = 2.199–4.301; *p* < 0.0001), larger households (OR = 2.02; 95% CI = 1.337–3.117; *p* = 0.0011) and those with higher monthly household income (OR = 3.39; 95% CI = 1.945–5.816; *p* < 0.0001) were more likely to have good knowledge of appropriate antibiotic use and antibiotic resistance. Furthermore, bad attitudes influenced participants' use of antibiotics without prescription (OR = 2.41; 95% CI = 0.432–4.05; *p* = 0.0009).

**Conclusion:**

This study exposes the drivers of inappropriate use of antibiotics at the household level, particularly in urban informal settlements. Policy interventions aimed at controlling the indiscriminate use of antibiotics in such settlements could improve the responsible use of antibiotics. Keywords: antibiotic resistance, informal settlements, Tamale, Ghana

## 1 BACKGROUND

The upsurge of antimicrobial resistance (AMR) remains a global challenge, putting at risk the progress made toward infectious disease prevention, treatment, and management,^1^, ^2^ hence seen as one of the top public health threats of the twenty‐first century.^3^ It has been projected that by the year 2050, AMR could cause about 10 million deaths annually if appropriate and timely interventions are not implemented.^1^, ^2^ Low and middle‐income countries (LMICs), especially Africa, which bears >90% of the global infectious diseases burden, will be the major recipient of the ravaging effect of AMR.^2^, ^3^

AMR, which used to be linked with healthcare facilities, is now common in informal settlements in many LMICs.^4^ Such informal settlements are characterized by high population density, poor sanitation, lack of consistent access to clean water, insecure residential status, and high participation in the informal economy.^4^, ^5^ These features promote the spread of infectious diseases and the demand for antibiotics.^4^, ^5^, ^6^ Antibiotics purchased without prescriptions and from unqualified vendors are common in these informal settlements. Although unacceptable, the economic status of these settlements permits residents to buy antibiotics in bits if they cannot pay for the complete course or from informal sources.^7^

The cost of antibiotic resistance to the global economy is significantly huge. In addition to death and disability, prolonged illness results in longer hospital stays, the need for more expensive medicines, and an increased financial burden on those affected. In 2019, an estimated 4.95 million deaths were associated with AMR at a rate of 27.3 deaths per 100,000 across all‐age death in western sub‐Saharan Africa.^3^ This reveals the urgent need to curb antibiotic resistance.

One of the major challenges confronting the fight against antibiotic resistance is understanding the knowledge gaps, attitudes, and practices (KAPs) that contribute to the use of antibiotics in LMICs at the household level, especially in areas where surveillance and data are scarce. This is critical to the development of practical interventions. To the best of our knowledge, no research work assessing the KAPs on antibiotic use and resistance of households in Ghanaian urban slums has been published. The study, therefore, assessed the relationship between KAPs and antibiotic use among households in urban informal settlements in the Tamale metropolis of Ghana.

## 2 METHOD

### 2.1 Study area

The study was conducted in two major informal settlements in the Tamale Metropolitan Distict in the Northern Region of Ghana between February and August 2022. The Metropolis lies between latitude 9°16 and 9°34 north and longitude 0°36 and 0°57 west.^8^, ^9^ The metropolis has one of the fastest‐growing populations with a current population of 374,744, of which 185,051 are males and 189,693 are females. There are about 35,408 households in the metropolis with an average of 11 members per household.^9^ The two major communities of the metropolis which are characteristic of an urban informal settlement per the metropolitan's demography were selected for this study. These communities were Dungu‐Asawaba and Moshe Zongo, characterized by many drugs or chemical shops and other informal sales of medicines, compounded with lack of safe and hygienic sanitary facilities.

### 2.2 Selection of household and participants

The households were selected using a multistage sampling method. First, we purposefully selected the two major suburbs which had the characteristics of urban informal settlement as described by Nadimpalli et al.^4^ The second stage involved a random selection of houses or housing units. In each suburb, major streets were used to divide the suburb into quadrants, and an approximately equal number of houses were sampled from each quadrant. Third, household each was randomly selected from each housing unit. Only households with at least a child <5 years old and an adult with knowledge of household healthcare practices were considered for random selection. From each suburb, 330 households were recruited, making a total of 660 for the study. The sample size was calculated using the Cochrane formula (*n* = *Z* ^2^ *P* (1 − *P*)/*d* ^2^).^10^ The confidence interval was 95%, the *Z* statistic was 1.96, and the *P* was 74.1% from previous studies,^11^ which gave a minimum sample size of 326.

### 2.3 Data collection procedure and study instrument

We searched through available literature and adopted our questionnaire to assess the KAP regarding antibiotic use and resistance among our study participants.^1^, ^5^, ^7^, ^12^, ^13^, ^14^, ^15^, ^16^, ^17^ The questionnaire had sociodemographic, knowledge, attitude, and practice sessions. We calculated the inter‐relatedness of the questions in each section using Cronbach's alpha. The reliability scores were 0.72, 0.65, and 0.5 for knowledge, attitude, and practice, respectively. Trained research assistants administered the questionnaire, and when appropriate, the questionnaire was translated into the respondents' native language.

The responses to the knowledge questions were either “True” or “False,” or “Yes” or “No,” and scored 1 for a correct response. We calculated the knowledge score (K‐score) as the number of correct answers out of the 10 questions. Knowledge score was categorized as good or poor based on the mean K‐score as described by Bulabula et al.^18^

For the attitude scores, each desired answer was scored 1 and the mean score was calculated for each respondent. Those who had an attitude score equal to or above the mean score were classified as having a good attitude and those below the mean score were recorded as bad attitude. For practices, each desired response was also scored 1.

### 2.4 Data management and analysis

The data were entered into Microsoft excel and later exported into STATA software version 13.1 (Stata) for all statistical analyses.

Data analysis of the KAP study included descriptive and analytic components. Descriptive analysis included frequencies, mean and SD for all scores after checking for their normal distribution. We reported frequencies of correct answers in the knowledge section and expected answers for the attitude and practice sections.

For the analytic component, the relationship between baseline characteristics, attitudes, and practice with K‐score using the chi‐square test. We also compare the proportions of desired attitude and practice between the K‐scores. Furthermore, we used logistic regression models to identify predictors of antibiotic self‐medication and assess the correlation between knowledge and sociodemographic characteristics. A *p* < 0.05 was considered statistically significant.

### 2.5 Ethical considerations

The study was approved by the Ethical Review Committee of the University for Development studies. The Tamale Metropolitan Health Directorate permitted us to conduct the research at the study sites. Ethical clearance was given by the University for Development Studies Institutional Review Board with reference UDS/RB/0121/22. Informed consent was obtained from all participants after explaining the study objectives, risks, benefits, right to refuse, and confidentiality. Participation was purely based on volunteerism. The identity and data of participants were kept confidential.

## 3 RESULTS

### 3.1 Characteristics of the respondents and households

The mean (SD) age of the household respondents was 28 (2.3) years. In terms of their educational attainment, 155 (23.5%) had no formal education, whereas 208 (31.5%), 218 (33.0%), and 79 (12.0%) had primary, secondary, and tertiary education, respectively. The household size ranges from 3 to 12 with 158 (23.9%) having a household size of 3–5. The most predominant household size was 6–8; 293 (44.4%) and 209 (31.7%) had more than 8 household members. The household respondents were predominantly females, 442 (67.0%). The monthly household income (MHI) in Ghana Cedis was less than 1000 in 244 (37.0%) households, whereas the remaining earned between 1000 and 3000 (Table 1).

**Table 1:** Household characteristics, attitudes, and practice of antibiotic use and antibiotic resistance of respondents versus knowledge level.

Characteristic	Variable	*N*	(%)	Knowledge score					
				Good		Poor			
				*n*	(%)	*n*	(%)	*χ* ^2^, df	*p*
Age	21–32	174	(26.4%)	76	(43.7%)	98	(56.3%)	9.562, 3	0.023
	33–43	173	(26.2%)	99	(57.2%)	74	(42.8%)		
	44–51	155	(23.5%)	87	(56.1%)	68	(43.9%)		
	52–64	158	(23.9%)	92	(58.2%)	66	(41.8%)		
Sex	Male	218	(33.0%)	107	(35.3%)	111	(64.7%)		0.115
	Female	442	(67.0%)	247	(62.7%)	195	(37.3%)		
Marital status	Single	46	(7.0%)	40	(87.0%)	6	(13.0%)		<0.001
	Married	614	(93.0%)	314	(51.1%)	300	(48.9%)		
Household size	3–5	158	(23.9%)	56	(35.4%)	102	(64.6%)	34.17, 2	<0.001
	6–8	293	(44.4%)	188	(64.2%)	105	(35.8%)		
	9 and above	209	(31.7%)	110	(52.6%)	99	(47.4%)		
Educational level	None	155	(23.5%)	25	(16.1%)	130	(83.9%)	196.7, 3	<0.001
	Primary	208	(31.5%)	86	(41.3%)	122	(58.7%)		
	Secondary	218	(33.0%)	183	(83.9%)	35	(16.1%)		
	Tertiary	79	(12.0%)	60	(75.9%)	19	(24.1%)		
Household income	Below 1000	244	(37.0%)	123	(50.4%)	121	(49.6%)	24.02, 3	<0.001
	1000–2000	173	(26.2%)	83	(48.0%)	90	(52.0%)		
	2000–3000	154	(23.3%)	79	(51.3%)	75	(48.7%)		
	Above 3000	89	(13.5%)	69	(77.5%)	20	(22.5%)		
Was this medicine prescribed to you/them at a health facility?									
	Yes	87	(29.9%)	62	(56.0%)	25	(44.0%)		<0.001
	No	204	(70.1%)	88	(51.8%)	116	(48.2%)		
Did any other person in this household use this same medicine									
	Yes	96	(33.0%)	41	(45.9%)	55	(54.1%)		0.002
	No	195	(67.0%)	122	(56.4%)	73	(43.6%)		
Attitude score	Good	266	(40.3%)	142	(53.4%)	124	(46.6%)		0.937
	Bad	394	(59.7%)	212	(53.8%)	182	(46.2%)		

### 3.2 Household history of antibiotic use

In all, 660 household interview responses were collected and analyzed. Two hundred and ninety‐one (44.1%) of the households reported antibiotic use episodes within the past month, within the study period, out of which 87 (29.9%) were prescribed and 204 (70.1%) were nonprescribed. The overall prevalence of unprescribed antibiotics used among households was 30.9% (204/660).

**Figure 1:**
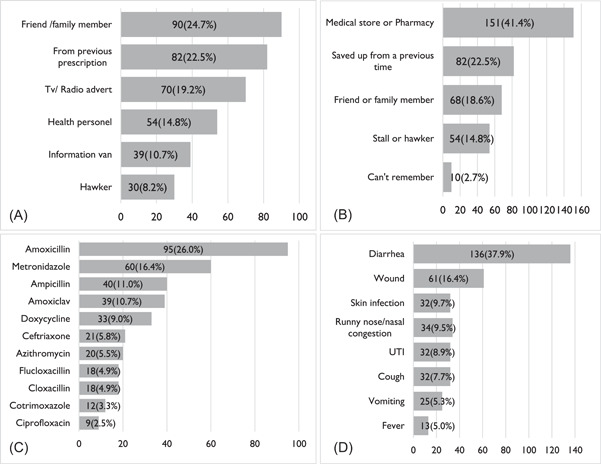
Household history of antibiotic use. (A) Source of information on which antibiotics to use. (B) Sources of antibiotics. (C) Types of antibiotics used. (D) Disease conditions for which antibiotics was used.

The history of antibiotic use per the households in this study covered reported cases by the enrolled adult, enrolled child (response provided by the enrolled adult), and any other adult household member. Enrolled adults represented 138/365 (37.8%), enrolled children were 120/365 (32.9%), and other adults of the households were 107/365 (29.3%). For those who obtained antibiotics without a prescription, information on which antibiotics to use was obtained from either a friend/family member 90 (24.7%), from a previous prescription 82 (22.5%), Tv/radio advert 70 (19.2%), health personnel 54 (14.8%), information van 39 (10.7%), or drug hawker 30 (8.2%) (Figure 1A). These antibiotics were obtained from a medical store or pharmacy 151 (41.4%), saved up from a previous time 82 (22.5%), friend or family member 68 (18.6%), drug hawker 54 (14.8%), and those who could not remember were 10 (2.7%) (Figure 1B).

Amoxicillin 95 (26.0%) was the most used antibiotic in the households followed by Metronidazole 60 (16.4%), Ampicillin 40 (11.0%), Amoxiclav 39 (10.7%), Doxycycline 33 (9.0%), Ceftriaxone 21 (5.8%), Azithromycin 20 (5.5%), Cloxacillin 18 (4.9%), Flucloxacillin 18 (4.9%), Cotrimoxazole 12 (3.3%), and Ciprofloxacin 9 (2.5%) (Figure 1C). Most of the household members took antibiotics because of diarrhea 136 (37.9%) and wound infections 59 (16.4%) as shown in Figure 1D.

### 3.3 Knowledge of antibiotic use and antibiotic resistance

**Figure 2:**
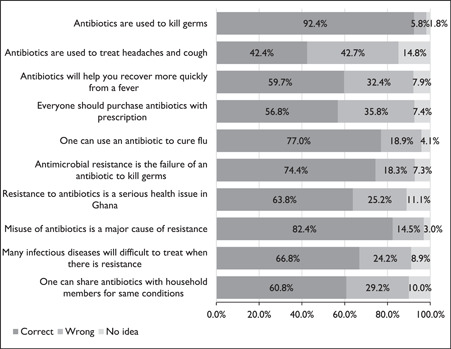
Proportions of responses to knowledge assessment on antibiotic use and antibiotic resistance.

More the half of the household respondents 354 (53.6%) had a good knowledge score of 6.2 (1.4) on antibiotic use and resistance. As shown in Figure 2, about 610 (92.4%) household respondents knew antibiotics are used to kill germs and 375 (56.8%) indicated antibiotics should be purchased with a prescription. Although 508 (77.0%) believed antibiotics can be used to cure flu, 280 (42.4%) others thought they can be used to treat headaches and cough. In addition, 394 (59.7%) knew that antibiotics will not help recover quickly from a fever. Four hundred and ninety‐one (74.4%) answered that AMR is the failure of an antibiotic to kill germs and 421 (63.8%) knew that resistance to antibiotics is a serious health challenge in Ghana. Additionally, 544 (82.4%) were aware that misuse of antibiotics is a major cause of resistance and 401 (60.8%) others revealed that one must not share antibiotics with household members for the same conditions. In all, a greater number of respondents 441 (66.8%) answered correctly that many infectious diseases will be difficult to treat when there is resistance to existing antibiotics.

### 3.4 Attitude toward antibiotic use and antibiotic resistance

**Figure 3:**
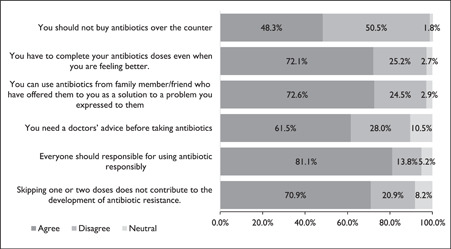
Proportions of responses to attitude assessment on antibiotic use and antibiotic resistance.

Of the 660 household respondents, 319 (48.3%) agreed that one should not buy antibiotics over the counter (OTC) and 406 (61.5%) agreed one needs a doctor's advice before taking antibiotics. Respondents agreed that one must complete your antibiotics doses even when you are feeling better 476 (72.1%) and only 162 (24.5%) disagree that one can use antibiotics from a family member/friend who has offered them to you as a solution to a problem you expressed to them. Even though a greater number of the respondents 558 (84.5%) agreed that infection with resistant germs can be life‐threatening, 535 (81.1%) agreed that everyone should responsibly use antibiotics. In addition, 468 (70.9%) of the respondents agreed that skipping one or two doses does not contribute to the development of antibiotic resistance (Figure 3).

### 3.5 Factors associated with antibiotic use and antibiotic resistance knowledge

Regarding knowledge, older respondents, female respondents, larger household size, respondents with higher educational attainment, and households with higher monthly income were more likely to have good knowledge of appropriate antibiotic use and antibiotic resistance (Table 2).

**Table 2:** logistic regression analysis of household respondent characteristics with knowledge of appropriate antibiotic use and antibiotic resistance and antibiotic self‐medication.

	Knowledge of antibiotic use and antibiotic resistance							Antibiotic use without prescription/self‐medication (*N* = 291)						
Characteristics	Good		Poor		OR	95% CI	*p*	No		Yes		OR	95% CI	*p*
	*n*	%	*n*	%				*n*	%	*n*	%			
Age														
21–32	76	(43.7%)	98	(56.3%)	1			73	(81.1%)	17	(18.9%)	1		
33–43	99	(57.2%)	74	(42.8%)	1.73	1.135–2.648	0.0135	65	(63.7%)	37	(36.3%)	0.41	0.205–0.787	0.0098
44–51	87	(56.1%)	68	(43.9%)	1.65	1.061–2.527	0.0273	47	(78.3%)	13	(21.7%)	0.48	0.232–1.001	0.0065
52–64	92	(58.2%)	66	(41.8%)	1.80	1.158–2.751	0.0086	19	(48.7%)	20	(51.3%)	0.22	0.0971–0.521	0.0003
Sex														
Male	77	(35.3%)	141	(64.7%)	1			48	(58.5%)	34	(41.5%)	1		
Female	277	(62.7%)	165	(37.3%)	3.07	2.199–4.301	<0.0001	39	(18.7%)	170	(81.3%)	0.16	0.09369–0.284	<0.0001
Household size														
3–5	56	(35.4%)	102	(64.6%)	1			19	(41.3%)	27	(58.7%)	1		
6–8	188	(64.2%)	105	(35.8%)	3.26	2.155–4.833	<0.0001	27	(18.6%)	118	(81.4%)	0.33	0.1610–0.650	0.0028
≥9	110	(52.6%)	99	(47.4%)	2.02	1.337–3.117	0.0011	41	(41.0%)	59	(59.0%)	0.99	0.4849–1.943	>0.9999
Educational level														
None	25	(16.1%)	130	(83.9%)	1			13	(18.6%)	57	(81.4%)	1		
Primary	86	(41.3%)	122	(58.7%)	3.67	2.217–6.163	<0.0001	21	(20.6%)	81	(79.4%)	1.14	0.5390–2.419	0.8463
Secondary	183	(83.9%)	35	(16.1%)	27.19	15.45–46.08	<0.0001	40	(40.4%)	59	(59.6%)	2.97	1.477–5.885	0.0026
Tertiary	60	(75.9%)	19	(24.1%)	16.42	8.166–30.74	<0.0001	13	(65.0%)	7	(35.0%)	8.14	2.733–23.14	<0.0001
Household income														
>1000	123	(50.4%)	121	(49.6%)	1			42	(34.1%)	81	(65.9%)	1		
1000–2000	83	(48.0%)	90	(52.0%)	0.91	0.6097–1.347	0.691	12	(19.4%)	50	(80.6%)	0.46	0.2254–0.931	0.0407
2001–3000	79	(51.3%)	75	(48.7%)	1.04	0.6926–1.552	0.9181	20	(29.9%)	47	(70.1%)	0.82	0.4392–1.529	0.6279
≥3000	69	(77.5%)	20	(22.5%)	3.39	1.945–5.816	<0.0001	13	(33.3%)	26	(66.7%)	0.96	0.4569–2.001	>0.9999
Sharing of antibiotics														
No	122	(62.6%)	73	(37.4%)	1			–	–	–	–	–	–	–
Yes	41	(42.7%)	55	(57.3%)	2.242	1.349–3.677	0.0017	–	–	–	–	–	–	–
Knowledge score														
Good	–	–	–	–	–	–	–	20	(15.6%)	108	(84.4%)	1		
Poor	–	–	–	–	–	–	–	67	(41.1%)	96	(58.9%)	3.77	2.101–6.602	<0.0001
Attitude score														
Good	–	–	–	–	–	–	–	63	(58.3%)	45	(41.7%)	1		
Bad	–	–	–	–	–	–	–	141	(77.0%)	42	(23.0%)	2.4	1.432–4.057	0.0009

Females had a 3.1 times higher chance of having a good knowledge of appropriate antibiotic use and antibiotic resistance (odds ratio [OR] = 3.07; 95% confidence interval [95% CI] = 2.199–4.301; *p* < 0.0001). Respondents from larger households and those with higher MHI were more than twice (OR = 2.02; 95% CI = 1.337–3.117; *p* = 0.0011) and more than thrice (OR = 3.39; CI = 1.945–5.816; *p* < 0.0001) likely to have good knowledge of appropriate antibiotic use and antibiotic resistance respectively. Similarly, respondents who had attained secondary and tertiary levels of education were, respectively, about 27 and 16 times more likely to have good knowledge, whereas those with a primary level of education were more than trice like to have poor knowledge (OR = 3.67; 95% CI = 2.217–6.163), compared with those with no formal education. We also detected that the older respondents had higher odds of having good knowledge of appropriate antibiotic use and antibiotic resistance (OR = 1.80; 95% CI = 1.158–2.751 *p* = 0.0086) than the younger respondents. In addition, household respondents with poor knowledge levels were two times more likely to share antibiotics with household members (OR = 2.242; 95% CI = 1.349–3.677; *p* = 0.0017) (Table 2).

### 3.6 Factors associated with antibiotic self‐medication (use of antibiotics without prescription)

The logistic regression showed that household respondents with high educational levels, poor knowledge, and bad attitude were more likely to use antibiotics without prescription (self‐medication), whereas female respondents, older respondents, those from small household sizes, and those with high MHI and bad attitudes were less likely to self‐medicate as shown in Table 2.

The older the household respondent, the higher their odds to self‐medicate (OR = 3.91; 95% CI = 1.388–10.16; *p* = 0.014). Persons with the highest form of education were eight times more likely to self‐medicate (OR = 8.14; 95% CI = 2.733–23.14; *p* < 0.0001). Those with bad attitudes were more than two times like to use antibiotics without prescription (OR = 2.41; 95% CI = 0.432–4.05; *p* = 0.0009) as seen in Table 2.

Females had an 84% reduction in odds (OR = 0.16; 95% CI = 0.09369–0.2834; *p* < 0.0001) and respondents from households with the size of 6–8 had a 67% reduction in odds (OR = 0.33; 95% CI = 0.1610–0.6501; *p* = 0.0028). In addition, respondents from households with MHI between Ghc1000 and Ghc2000 were less likely (OR = 0.46; 95% CI = 0.2254–0.9308; *p* = 0.0407) to self‐medicate with antibiotics. Furthermore, older respondents had 78% reduced odds (OR = 0.22; 95% CI = 0.0971–0.521; *p* = 0.0003) to use antibiotics without a prescription (Table 2).

## 4 DISCUSSION

This study focused on the drivers of antibiotic use at the household level in urban informal settlements in the Tamale Metropolitan District of the Northern Region of Ghana. The 291 (44.1%) households that reported the use of antibiotics within a month before this study was relatively higher compared with other studies that reported on antibiotic use in the general population.^4^, ^7^, ^17^, ^19^ However, this is lower than the 66% and 73% of respondents elsewhere in Ghana,^15^ the 81.25% in Pakistan,^20^ and 60.7% in Cameroun.^21^ This observation suggests that the use of antibiotics is common among households in urban informal settlements in Ghana. In assessing the sources of unprescribed antibiotics, we realized that 41.2% purchased their antibiotics from licensed chemical stores and pharmacies. This highlights the critical role these agencies play in antibiotic stewardship in the country. Interestingly, 17 (8.3%) households received information on the use of antibiotics from drug hawkers/peddlers, and 30 (14.7%) purchased antibiotics from the same. The sale of antibiotics through informal channels is characterized by counterfeit drugs^22^ and has been associated with the inappropriate use of antibiotics.^2^ This route may be a threat to the health of consumers and could defeat the goals of antibiotic stewardship if the necessary attention is not given to it.^22^ The practice of using leftover antibiotics and using previous prescriptions must be discouraged through community‐based education.

In our study, amoxicillin was the most frequently used antibiotic among households just as reported in other studies which assessed community usage of antibiotics.^5^, ^7^, ^15^, ^23^, ^24^ This should inform policymakers about the increasing use of such broad‐spectrum antibiotics at the community level and the need to control their use. We also found out that 30.9% of households that used antibiotics purchased them without a prescription. This rate is similar to the 32% reported by Mensah et al.^25^ and the 36% by Kretchy et al.^7^ in rural communities in Ghana. Even though antibiotic self‐medication has been reported as a widespread phenomenon in some LMICs, Do et al.^1^ studies have reported a low prevalence in Mozambique (8.0%), Thailand (3.9%), and South Africa (1.2%).^1^ In Ghana, the availability of a wide range of antibiotics in the open market,^1^, ^26^ the extensive OTC dispensing of drugs,^26^ and the financial gains of medicine sellers propel the community demand for antibiotics. Access to antibiotics without prescription is common in many LMICs.^1^, ^2^, ^4^, ^17^, ^18^, ^26^, ^27^ This has resulted in the indiscriminate use of antibiotics and its associated rise in antibiotic resistance in such countries.^4^, ^5^

Antibiotic use without prescription (self‐medication) was associated with older respondents, those with higher educational levels, low MHI, larger households, and poor knowledge. Similar to this study, other studies have found an association between socioeconomic status and antibiotic self‐medication.^18^, ^28^ Households with low MHI have reduced purchasing power. This affects their ability to pay for quality healthcare and full‐course of quality drugs that may be prescribed at the hospital.^2^ They, therefore, opt for OTC where they can buy drugs in bits.^1^, ^2^, ^26^ Knowledge of the appropriate use of antibiotics may affect a household member's decision to self‐medicate. In Pakistan, Gillani et al.,^29^ in Lebanon, Jamhour et al.,^30^ and in Ghana, Afari‐Asiedu et al.^2^ and Kretchy et al.^7^ reported that self‐medication with antibiotics is associated with knowledge and that persons with low knowledge were more likely to self‐medicate. Another inappropriate use of antibiotics is the sharing of antibiotics and dependence on previous prescriptions to purchase an antibiotic for a new or similar health condition. Household respondents with poor knowledge levels were two times more likely to share antibiotics with household members.

Assessing the knowledge level, the 354 (53.6%) household respondent who had good knowledge is comparable to a report by Taha et al.,^28^ where 54.9% of the participants had good knowledge of antibiotic use and antibiotic resistance. This, however, is higher than other studies, which reported 43.9% in Lebanon^31^ and 45% in Palestine.^28^ Even though the highest educational level among the household respondent was secondary school, more than half had good knowledge of antibiotic use and resistance. This supports the fact that there are many means through which they acquire information. Specifically, most of the household respondents received information from family and friends (24.5%), previous prescriptions (22.5%), and television and radio (19.1%). Other studies have reported a variety of sources from which participants received information besides formal education.^1^, ^2^, ^17^, ^18^, ^26^ This presents policymakers with the opportunity to rightly inform households on appropriate antibiotic use.

Several factors have been associated with knowledge of antibiotic use and resistance.^4^, ^15^, ^17^, ^27^, ^31^ In this study, respondents with higher levels of education, and wealthy households were more likely to have good knowledge than the uneducated ones. Other studies have documented a significant association between knowledge and education.^15^, ^16^, ^17^ This could be attributed to the fact that education is a determinant of health literacy which includes antibiotic use and resistance.^17^ In addition, the higher chance of female household respondents having good knowledge is not coincidental as in the study area mothers are mostly caregivers of the household, especially to the children.^32^ Respondents from households with larger household size are more likely to have good knowledge of appropriate antibiotic use and antibiotic resistance than those with small household size. Fabrigar et al.^33^ reported in their experimental studies that high levels of knowledge underline people's attitudes, behavior, and choice. Therefore, to curb inappropriate use of antibiotics at the household level, targeted educational intervention may improve attitudes and practices toward antibiotic use. We conclude that bad attitude, poor knowledge, larger household size, low MHI, and higher formal educational level were drivers of antibiotic use without prescriptions in our study area.

The strength of the study is that it is a comprehensive assessment of the factors associated with antibiotic use at the household level. This is the first compressive study at the household level. been published in Ghana.

The limitations of the study are, first, antibiotic use was based on recall of past events; thus, the accuracy and volume of information may have been influenced. Second, the KAP analysis was based on the household respondent's responses and not all individuals in each household.

## 5 CONCLUSION

AMR is becoming a global phenomenon and if pragmatic steps are not taken, it will make the treatment or prevention of existing and emerging infectious diseases difficult. The urban informal settlement is characterized by factors that drive irresponsible antibiotic use. At the household level in these settlements, respondents that were females, with larger household sizes, higher educational levels and households with higher HMI were more likely to have good knowledge of the appropriate antibiotic use and antibiotic resistance. However, older respondents, those with high educational levels, low HMI, larger households, and poor knowledge were more likely to use antibiotics without prescription (self‐medication). This study exposes the routes and drivers of inappropriate use of antibiotics at the household level, particularly in urban informal settlements, particularly in the Tamale metropolis. Policy interventions aimed at controlling the indiscriminate use of antibiotics in such settlements could improve the responsible use of antibiotics. Future study should be conducted to assess the influence of self‐medication with antibiotic use on the antibiotic succeptibility profile of bacteria isolated from members in the study area.

## AUTHOR CONTRIBUTIONS

**Ezekiel K. Vicar**: Conceptualization; formal analysis; writing—original draft; writing—review & editing. **Williams Walana**: Formal analysis; writing—original draft; writing—review & editing. **Augustina Mbabila**: Conceptualization; investigation; methodology; writing—review & editing. **George K. Darko**: Investigation; methodology; writing—review & editing. **Kwame Opare‐Asamoah**: Conceptualization; writing—original draft; writing—review & editing. **Saeed F. Majeed**: Data curation; formal analysis; writing—review & editing. **Mauvina Obeng‐Bempong**: Investigation; methodology; writing—review & editing.

## CONFLICT OF INTEREST STATEMENT

The authors declare no conflict of interest.

## TRANSPARENCY STATEMENT

The lead author Ezekiel K. Vicar affirms that this manuscript is an honest, accurate, and transparent account of the study being reported; that no important aspects of the study have been omitted; and that any discrepancies from the study as planned (and, if relevant, registered) have been explained.

## Data Availability

Data will be made available upon reasonable request from the corresponding author.
